# Knowledge regarding Alzheimer's Disease among College Students of Kathmandu, Nepal

**DOI:** 10.1155/2020/6173217

**Published:** 2020-01-09

**Authors:** Kushalata Baral, Maginsh Dahal, Shneha Pradhan

**Affiliations:** ^1^Department of Public Health, Nobel College, Pokhara University, Nepal; ^2^Department of Public Health, Asian College for Advance Studies, Purbanchal University, Nepal

## Abstract

**Introduction:**

Alzheimer's, a neurodegenerative disease, is becoming a growing burden and the leading cause of disability among older people, and there is no cure for it. It is set to be the biggest killer among the growing elderly population. The aim of this study was to assess the knowledge of Alzheimer's disease among college students in Kathmandu metropolitan city.

**Methods:**

This was a descriptive cross-sectional study among 385 randomly selected bachelor students of Kathmandu metropolitan city. The questionnaire included 2 sections. Section I addressed the sociodemographic characteristics of the participants. Section II addressed or covered the Alzheimer's Disease Knowledge Scale (ADKS) test. ADKS contains a set of 30 items, with true and false options. 1 point was given for the correct answer and 0 for the incorrect answer. The final sum was then the total score of the participant. Frequency, percentage, mean, and standard deviation were calculated, and the chi-square test was used to measure the association between two categorical variables.

**Results:**

The mean ADKS (Alzheimer's Disease Knowledge Scale) score is 15.45 ± 2.95 with the lowest and highest mean total scores of 8 and 26, respectively. 49.5% of the respondents scored above the mean. The number of male and female respondents who scored above the mean is 68 and 95, respectively, with *p* value 0.71 and odds ratio 0.922. There is no association between gender and knowledge level. Gender seemed to have no effect on the knowledge about Alzheimer's disease on the basis of the Alzheimer's Disease Knowledge Scale (ADKS). However, science students had comparatively better knowledge about disease than management students. The mean score of science and management is 15.9 and 15.04, respectively, with *p* value 0.004. There is association between knowledge score and faculty.

**Conclusion:**

This study concluded that the knowledge level of college students on Alzheimer's disease is below moderate. The findings concluded that there is association between faculty and knowledge score.

## 1. Introduction

Alzheimer's disease is defined as the degenerative disease of the brain resulting in progressive memory loss, impaired thinking, deterioration, and changes in personality and mood [1]. It includes deterioration of language, comprehension, memory, and thinking and learning capability [2]. The term Alzheimer was first coined by a German physician, Alios Alzheimer, in 1915 [3]. The WHO mentioned Alzheimer's disease as the most common cause of dementia; however, not all dementia is a result of Alzheimer's [4]. Alzheimer's is becoming a growing burden and the leading cause of disability among older people, and there is no cure for it [5]. It is set to be the biggest killer among the growing elderly population [6].

Alzheimer's disease worsens over time [7]. It is a progressive disease, where dementia symptoms gradually worsen over a number of years [8]. In its early stages, memory loss is mild, but with late-stage Alzheimer's disease, individuals lose the ability to carry on a conversation and respond to their environment [4]. Those with Alzheimer's disease live an average of eight years after their symptoms become noticeable to others, but survival can range from 4 to 20 years, depending on age and other health conditions [9].

About 70 percent of the risk is believed to be genetic with many genes usually involved. Other risk factors include a history of head injuries, depression, or hypertension [4]. The disease process is associated with plaques and tangles in the brain. Initial symptoms are often mistaken for normal ageing [10]. Mental and physical exercise and avoiding obesity may decrease the risk of Alzheimer's disease [11]. There are no medications or supplements that decrease the risk [6]. No treatments can stop or reverse its progression, though some may temporarily improve symptoms [9]. The affected people increasingly rely on others for assistance, often placing a burden on the caregiver; the pressures can include social, psychological, physical, and economic elements [12].

The number of people with Alzheimer's disease has been increasing among the elderly population in Nepal since the past few years. AD is the most common form of dementia, a group of disorders that impairs mental functioning of an individual [2]. The causes of Alzheimer's disease are poorly understood [9]. And the awareness about this disease and the social and health needs of the patients is still low, and many families in Nepal seemed to be compelled to face it alone [3]. Thus, the study tries to find out the knowledge of Alzheimer's disease among college students in Kathmandu metropolitan city.

## 2. Materials and Methods

The descriptive cross-sectional study using quantitative methods was conducted on seven undergraduate colleges affiliated to Pokhara University of Kathmandu. The sample size was 385 and was calculated using 50% prevalence with confidence limit of 95%. The stratified random sampling technique was used for obtaining the number of colleges, and the census method was used for selecting the study population. A self-administered questionnaire was used for data collection, which also included the Alzheimer's Disease Knowledge Scale consisting of 30 knowledge assessment items after pretesting on 10% of the sample, i.e., 38 of the respondents with similar characteristics. The questionnaire included 2 sections. Section I addressed the sociodemographic characteristics of the participants. Section II addressed or covered the Alzheimer's Disease Knowledge Scale (ADKS) test. ADKS contains a set of 30 items, with true and false options. 1 point was given for the correct answer and 0 for the incorrect answer. The final sum was then the total score of the participant. Content validity of the instrument was obtained from literature review. Random error was reduced by selecting adequate sample size. Bias and error were reduced by selecting a sound study design. Reliability testing and consistency checking of data were done. Ethical clearance was taken from the Institutional Review Committee. The college authorities were contacted for permission to conduct the study. Data collection was conducted only after verbal consent of the participants. Privacy and confidentiality of the collected information were ensured through the use of anonymous data collection tools. Data entry was done in “Statistical Package for the Social Sciences” IBM statistics version 20.0. Under descriptive summary statistics of data, frequency and percentage were calculated. Additionally, mean and standard deviation were calculated, and the chi-square test was used to measure the association between two categorical variables.

## 3. Results

In Table 1, among a total of 385 respondents, more than half belonged to the age group 21-25 years, with 58.4% female. Hindu respondents were 91.7%, followed by Brahmin (36.9%). Management students were 62.3%.

Table 2 demonstrates the 30 statements of the Alzheimer's Disease Knowledge Scale, domain each statement falls under, correct answer of each statement, number of correct responses, mean, and standard deviation. The domains are as follows:

- LI = life impact
- RF = risk factors
- CO = course
- C = caregiving
- TM = treatment and management
- SY = symptoms
- AD = assessment and diagnosis

The mean ADKS score is 15.45 ± 2.95 with the lowest and highest mean total scores of 8 and 26, respectively. 49.5% of the respondents scored above the mean.

In Table 3, the number of male and female respondents who scored below the mean is 66 and 100, respectively. And the number of male and female respondents who scored above the mean is 68 and 95, respectively, with *p* value 0.71 and odds ratio 0.922. There is no association between gender and knowledge level. The number of male and female respondents who scored below the mean is 58 and 108, respectively. And the number of male and female respondents who scored above the mean is 85 and 78, respectively, with *p* value 0.002 and odds ratio 0.493. There is association between faculty and knowledge level.

In Table 4, the mean score of male and female is 15.7 and 15.28, respectively, with *p* value 0.2. There is no association between knowledge score and gender. The mean score of science and management is 15.9 and 15.04, respectively, with *p* value 0.004. There is association between knowledge score and faculty.

## 4. Discussion

This study focused on bachelor-level college students in order to assess knowledge level on Alzheimer's disease.

The mean ADKS score was 15.45 (SD = 2.95) with the lowest mean total score 8 and highest mean total score 26. 49.5% of the respondents scored above the mean. 13 out of 385 respondents had more than 20 correct responses on the scale. In a study among college students in Iowa, USA, the mean on the ADKS was 20.78 (SD = 4.24) with the lowest mean score 2 and the highest mean score 29. 114 out of 200 respondents had more than 20 correct responses on the scale [13]. This may be due to the level of knowledge difference between American students and Asian students.

In our study, the mean age of the respondents was 20.63 ± 1.629 years old, where 41.6% were male and 58.4% were female. The mean score of male and female respondents is 15.7 and 15.28, respectively, with *p* value 0.2. In a study among undergraduate students of health and social care in Norway, the mean age of the students was 25.68 (SD = 5.91) years, and 84% were female. Independent *t*-tests demonstrated no significant difference in ADKS mean scores between genders. The mean score of male students was 23.20, and the mean score of female students was 23.57 [14].

In our study, only 0.95% of the respondents had at least one family member with AD or a related disorder with a mean ADKS score of 15. This shows that there is no significant difference in the knowledge score as compared to those who did not have at least one family member with a mean of 15.4. In a study in individuals from the USA with at least 1 family member with AD or a related disorder, 41% of the sample was more knowledgeable with a mean of 22.66 than those who did not have an affected family member with a mean of 20.81 [9]. This may be because of the high prevalence of diseases in the USA.

In our study, 58.7% of the respondents were aware that memory loss is the primary symptom of AD. 47.1% of the respondents said that AD had no cure. 33.7% of the respondents said that AD causes mental and physical decline. 93.3% of the respondents had well known the fact that AD is not communicable. In a study among Vietnamese American, most of the respondents, i.e., 96.8%, were aware of the fact that the primary symptom of AD is memory loss. 44.2% said that there is no cure for AD. 67.4% of the respondents said that AD causes both physical and mental decline, and 68.4% of the respondents said that AD is not communicable [15].

In our study, the mean score of science and management is 15.9 and 15.04, respectively, with *p* value 0.004. This shows that there is no significant difference between knowledge levels of science students and those in a different background. In a study conducted in Vietnam, the respondents in health care education showed higher levels of knowledge than those in a different background [15]. In our study, 45.9% of the respondents out of 385 believed that genetics is a very important risk factor and 44.7% of respondents believed that stress is very important in increasing AD risk. In the study in Copenhagen, 16% of the respondents out of 411 supported genetics as a very important risk factor and 20% respondents believed that stress is very important in increasing AD risk [16]. In our study, eating healthy diet (9.4% of the respondents believed), keeping mentally active (33.4% believed), and taking vitamins/dietary supplements (7.6% of the respondents believed) could be useful in the prevention of AD. In the study in the United States, eating healthy diet (40% of the respondents believed), keeping mentally active (47% believed), and taking vitamins/dietary supplements (47% of the respondents believed) could be useful in the prevention of AD [17].

## 5. Conclusion

This study concluded that the knowledge level of college students on Alzheimer's disease is below moderate. There were students who had not even heard about Alzheimer's disease. And the ones who had heard about Alzheimer's disease possessed low level of knowledge regarding the disease.

## 6. Recommendation

Alzheimer's disease can be included in the academic curriculum in order to provide students with basic information about the disease. 1/7 of the students had not even heard about AD. So, mass media and social organizations can be utilized to disseminate the information about AD in the community.

## Figures and Tables

**Table 1:** Demographic characteristics of the respondents (*n* = 385).

Variables	Frequency
Age	
17-20	191 (49.6%)
21-25	194 (50.45)
Gender	
Male	160 (41.6%)
Female	225 (58.4%)
Religion	
Hindu	353 (91.7%)
Buddhist	22 (5.7%)
Christian	6 (1.6%)
Muslim	41 (1%)
Ethnicity	
Brahmin	142 (36.9%)
Chhetri	138 (35.8%)
Dalit	5 (1.3%)
Janajati	83 (21.5%)
Others	17 (4.4%)
Marital status	
Married	16 (4.2%)
Unmarried	369 (95.8%)
Faculty	
Science	145 (37.7%)
Management	240 (62.35)

**Table 2:** ADKS measurement on the basis of the respondent's response (*n* = 385).

SN	Statements		Domain	Correct answer	Number	Mean	SD
1.	People with Alzheimer's disease are particularly prone to depression.		LI	True	249	0.76	0.430
2.	It has been scientifically proven that mental exercise can prevent a person from getting Alzheimer's disease.		RF	False	117	0.36	0.479
3.	After symptoms of Alzheimer's disease appear, the average life expectancy is 6-12 years.		CO	True	115	0.35	0.478
4.	When a person with Alzheimer's disease becomes agitated, a medical examination might reveal other health problems that caused the agitation.		AD	True	210	0.64	0.481
5.	People with Alzheimer's disease do best with simple instructions giving one step at a time.		C	True	164	0.50	0.501
6.	When people with Alzheimer's disease begin to have difficulty taking care of themselves, caregivers should take over right away.		C	False	76	0.23	0.422
7.	If a person with Alzheimer's disease becomes alert and agitated at night, a good strategy is to try to make sure that the person gets plenty of physical activity during the day.		C	True	226	0.69	0.464
8.	In rare cases, people have recovered from Alzheimer's disease.		CO	False	135	0.41	0.493
9.	People whose Alzheimer's disease is not yet severe can benefit from psychotherapy for depression and anxiety.		TM	True	203	0.62	0.487
10.	If trouble with memory and confused thinking appears suddenly, it is likely due to Alzheimer's disease.		AD	False	182	0.55	0.498
11.	Most people with Alzheimer's disease live in nursing homes.		LI	False	228	0.69	0.462
12.	Poor nutrition can make the symptoms of Alzheimer's disease worse.		TM	True	223	0.68	0.468
13.	People in their 30s can have Alzheimer's disease.		RF	True	159	0.48	0.500
14.	A person with Alzheimer's disease becomes increasingly likely to fall down as the disease gets worse.		CO	True	208	0.63	0.483
15.	When people with Alzheimer's disease repeat the same question or story several times, it is helpful to remind them that they are repeating themselves.		C	False	127	0.39	0.488
16.	Once people have Alzheimer's disease, they are no longer capable of making informed decisions about their own care.		C	False	128	0.39	0.488
17.	Eventually, a person with Alzheimer's disease will need 24-hour supervision.		CO	True	197	0.60	0.491
18.	Having high cholesterol may increase a person's risk of developing Alzheimer's disease.		RF	True	137	0.42	0.494
19.	Tremor or shaking of the hands or arms is a common symptom in people with Alzheimer's disease.		SY	False	141	0.43	0.496
20.	Symptoms of severe depression can be mistaken for symptoms of Alzheimer's disease.		AD	True	217	0.66	0.475
21.	Alzheimer's disease is one type of dementia.		AD	True	216	0.66	0.476
22.	Trouble handling money or paying bills is a common early symptom of Alzheimer's disease.		SY	True	180	0.55	0.499
23.	One symptom that can occur with Alzheimer's disease is believing that other people are stealing one's things.		SY	True	151	0.46	0.499
24.	When a person has Alzheimer's disease, using reminder notes is a crutch that can contribute to decline.		TM	False	129	0.39	0.489
25.	Prescription drugs that prevent Alzheimer's disease are available.		RF	False	148	0.45	0.498
26.	Having high blood pressure may increase a person's risk of developing Alzheimer's disease.		RF	True	177	0.54	0.499
27.	Genes can only partially account for the development of Alzheimer's disease.		RF	True	181	0.55	0.498
28.	It is safe for people with Alzheimer's disease to drive, as long as they have a companion in the car at all times.		LI	False	202	0.61	0.488
29.	Alzheimer's disease cannot be cured.		TM	True	134	0.41	0.492
30.	Most people with Alzheimer's disease remember recent events better than things that happened in the past.		SY	False	126	0.38	0.487
Sum_ADKS	Number	Minimum	Maximum		Mean	SD	
	329	8	26		15.45	2.95	

**Table 3:** Association between knowledge levels on Alzheimer's and gender and faculty of students.

Variables	Below the mean (8–15)	Above the mean (16-26)	*p* value	Odds ratio
Gender				
Male	66	68	0.71	0.922
Female	100	95		
Total	166	163		
Faculty				
Science	58	85	0.002	0.493
Management	108	78		
Total	166	163		

**Table 4:** Association between knowledge score on Alzheimer's and gender and faculty of students.

Variables	Number	Mean	SD	*p* value
Gender				
Male	134	15.7	3.15	0.2
Female	195	15.28	2.80	
Faculty				
Science	143	15.9	2.76	0.004
Management	186	15.04	3.03	

## Data Availability

The SPSS data used to support the findings of this study are available from the corresponding author upon request.
